# Convolutional-de-convolutional neural networks for recognition of surgical workflow

**DOI:** 10.3389/fncom.2022.998096

**Published:** 2022-09-07

**Authors:** Yu-wen Chen, Ju Zhang, Peng Wang, Zheng-yu Hu, Kun-hua Zhong

**Affiliations:** ^1^Chongqing Institute of Green and Intelligent Technology, Chinese Academy of Sciences, Chongqing, China; ^2^Southwest Hospital, Third Military Medical University, Chongqing, China

**Keywords:** neural networks, convolutional-de-convolutional, transfer learning, surgical workflow, deep learning

## Abstract

Computer-assisted surgery (CAS) has occupied an important position in modern surgery, further stimulating the progress of methodology and technology. In recent years, a large number of computer vision-based methods have been widely used in surgical workflow recognition tasks. For training the models, a lot of annotated data are necessary. However, the annotation of surgical data requires expert knowledge and thus becomes difficult and time-consuming. In this paper, we focus on the problem of data deficiency and propose a knowledge transfer learning method based on artificial neural network to compensate a small amount of labeled training data. To solve this problem, we propose an unsupervised method for pre-training a Convolutional-De-Convolutional (CDC) neural network for sequencing surgical workflow frames, which performs neural convolution in space (for semantic abstraction) and neural de-convolution in time (for frame level resolution) simultaneously. Specifically, through neural convolution transfer learning, we only fine-tuned the CDC neural network to classify the surgical phase. We performed some experiments for validating the model, and it showed that the proposed model can effectively extract the surgical feature and determine the surgical phase. The accuracy (Acc), recall, precision (Pres) of our model reached 91.4, 78.9, and 82.5%, respectively.

## Introduction

Computer-assisted surgery (CAS) emerged in the twentieth century, which means that computer technology is used to guide and assist surgeons. The application (Garg et al., 2005) provides decision-making support and planning tools in the preoperative. Intraoperative computer assistance includes robotic surgical system (Dergachyova, 2018), image guidance and navigation (Peters, 2006), augmented reality and visualization (Kersten-Oertel et al., 2013). Postoperative assistance provides tools to analyze executed procedures and results, as well as to improve and optimize (Schumann et al., 2015). Despite all the advance and valuable assistance, the seamless integration of computer-aided equipment with operating room (OR) and surgical procedures has not yet been achieved. Existing ORs contain a set of unrelated independent systems and devices, most of which appear in isolation, disabling proper communication and interaction (Hübler et al., 2014). Current computer-aided equipment facilitates a number of individual surgical tasks, but their lack of synchronization with the surgical process hampers the work and resource management of the surgical team. It leads to higher stress levels (Agarwal et al., 2006), frequent misunderstandings among surgical staffs, resulting in risks and delays, as well as inefficient surgical groups that incur excessive costs for hospitals (Macario, 2010).

Context-aware Computer-assisted surgery (CA-CAS) has powerful artificial intelligence that understands or perceives the needs of clinicians. It should always be aware of the events that occur, the actions performed, and the current state by tracking the surgical procedure and constantly observing the surgical site. Examples of applications are: optimization of the surgical procedure (Franke et al., 2013; Guédon et al., 2016), prediction of the remaining time of surgery (Bhatia et al., 2007), intraoperative assistance (Nessi et al., 2015; Fard et al., 2016), automatic generation of surgical reports (Agarwal et al., 2006). A large number of studies have focused on IntelliSense intraoperative aids to reduce the pressure on surgeons and facilitate the surgical process (Meng et al., 2021; Liu et al., 2022). Automatic recognition of surgical procedures is an important part of this. Recognizing surgical procedures is a prerequisite for CAS applications. The study on this subject began about 10 years ago. Despite the great progress made, it remains a relatively new area that inspires scientists and clinicians to inspire. Due to the lack of automatic recognition, most applications use manual label of surgical activities, which is a very tedious and time-consuming process.

Today, artificial intelligence and deep learning technologies have developed rapidly (Li et al., 2017; Liu et al., 2020; Zhong et al., 2021; Fan et al., 2022) and have been successfully applied in many different fields, including image labeling, natural language modeling, text generation, image labeling, natural language modeling, text generation, classification (Zheng et al., 2021), medical care (Zhang et al., 2020, 2021), web service QoS prediction (Wu et al., 2022), and risk assessment (Deng et al., 2022). In most cases, their performance is superior to that of traditional machine learning methods. Comprehensive and accurate training data have been playing an important role in machine learning. The quantity and quality of data have become an important factor. The size of the massive data sets that serve as a basis for the training of deep learning model, such as the famous ImageNet (Deng et al., 2009), Microsoft COCO (Deng et al., 2009), the recently released Google’s OpenImages (Krasin et al., 2017; Kuznetsova et al., 2020), and YouTube-8M (Abu-El-Haija et al., 2016; YouTube-8M Dataset, 2018), is self-evident. They contain millions of samples representing thousands of categories. Unfortunately, sometimes learning tasks have to be carried out in an area of interest expressed by a small group of data, such as the field of surgery. A variety of constraints hinder proper data collection: Ethical approvals, the consent of patients and medical personnel, the limited number of cases, the installation of expensive data acquisition equipment, and time-consuming manual annotations that require medical experience. In these cases, the methods of transfer learning may play a role. To a large extent, transfer learning involves the use of methods from resources in other areas of interest, where data may be distributed differently and located in different feature spaces, thus improving the learning of the target task. Depth models make it easy to transfer knowledge of one network to another. Transfer learning is a knowledge transfer technology that is currently widely used with convolution neural networks (CNN) for tasks related to visual content, which benefits from a large number of free datasets. It is also widely used in speech and language processing (Huang et al., 2013), document classification (Dai et al., 2007), sentiment analysis (Glorot et al., 2011), and other sequence analysis tasks.

Therefore, in this paper, we proposed an unsupervised method for training Convolutional-De-Convolutional (CDC) networks to sort surgical workflow frames, which are simultaneously rolled out in space (for semantic abstraction) and temporal convolution (for frame-level resolution). It has unique property in modeling the spatio-temporal interactions between high-level semantics in space and fine-grained action dynamics in time. Specifically, the CDC has to extract features related to understanding the surgical workflow. The knowledge learned from the task is encoded into the weight matrix of the internal parameters of the representation layer. Then the Convolutional-De-Convolution network is fine-tuned to classify the surgical phase.

The contributions of this paper are summarized as follows:

• We proposed a model that can solve the problem of annotating data deficiency in medical field by using the transfer learning method.
• We used a CDC network to recognize the surgical workflow because of its property of spatio-temporal interactions in training.
• We try to achieve intelligent detection of surgical video phase at a low cost. Finally, based on M2CAI 2016 challenge dataset, we performed experiments for validating the model. It shows a good performance compared with other methods.

This paper is organized as follows: Section II presents related work. We summarize methodology and the proposed models in section III. In section IV, we present the experiment and result of our method. In section V, we discuss conclusions and suggestions for future research.

## Related work

The OR’s understanding of surgical activities is a new field of research. Surgical workflow identification is closely related to multi-target tracking. Wang et al. (2022) proposed a General Recurrent Tracking Unit (RTU++), which can be flexibly plugged into other trackers, to score track proposals by capturing long-term information. And the experiments showed the generalization ability of RTU++ trained by simulated data in various scenarios. Under the specific limitations and difficulties implied by the surgical environment, only a few jobs deal directly with the application. Since the problem of surgical process identification is a multidisciplinary problem, we have decided to propose different related fields. Surgical phase recognition is similar to time action recognition. We start with a brief introduction to literatures on temporal action recognition. Then, we will focus on the internal approval of the operation.

### Temporal action recognition

Gaidon et al. (2011, 2013) introduced temporally action recognition in untrimmed videos, focusing on limited actions such as “drinking and smoking” (Calder and Siegel, 2009) and “opening the door to sit down” (Laptev and Perez, 2007). Later, researchers worked on building large datasets, including complex action categories such as THUMOS (Mexaction2, 2013), as well as datasets focused on fine-grained actions (Sigurdsson et al., 2016a,b) or high-level semantics activities (Heilbron et al., 2015). Recently, deep learning methods have shown better performance in localizing action instances. Franke et al. (2013) presented a temporal action proposal system based on Long-Short Term Memory (LSTM); Yeung et al. (2018) provided the MultiTHUMOS dataset of each frame multi-label annotations, and a LSTM network is defined to model multiple input and output connections; Shou et al. (2016) introduced a 3D CNN framework (S-CNN) based on end-to-end segmentation, which is superior to other RNN-based methods by capturing spatio-temporal information simultaneously. However, S-CNN lacks the ability to accurately predict time resolution and localize the exact time boundary of an action instance. In Shou et al. (2017), they proposed a CDC network for precise temporal action localization of untrimmed video, which provides a new CDC filter that can simultaneously perform spatial down-sampling (for spatio-temporal semantic abstraction) and temporal up-sampling (for precise time positioning). In this paper, we will use the CDC network structure to recognize the surgical phase by transfer learning. Details are described in the next section. Yang et al. (2018) proposed a Frame Segmentation Network (FSN), which placed a temporal CNN on top of the 2D spatial CNNs, and can make dense predictions at frame-level for a video clip using both spatial and temporal context information.

### Surgical phase recognition

Mackenzie et al. (2001) were among the first to propose the creation of a process model. In Mackenzie et al. (2001), it is based on structured multi-level decomposition that describes the surgical action performed during surgery. In the same year, Jannin et al. (2001) also proposed a neural process model based on Uniform Mark-up Language decomposition. Subsequently, the concept of surgical workflow was introduced. Neumuth et al. (2006) proposed the concept of the general methodology described in the acquisition process from surgical intervention, clinical and technical analysis, and automatic processing of workflow schemes can drive a workflow management system as the future of OR process control. Klank et al. (2008) used the evolutionary reinforcement learning to classify the laparoscopic cholecystectomy into 6 stages for the first time, with an Acc rate of about 50%. Klank et al. (2008) presented a method that based on Hidden Markov Model (HMM) and dynamic time warping algorithm (DTW) to perform a dimensionality reduction on image features by using additional information about tool usage for recognition of surgical workflow of laparoscopic video, the Acc of phase detection is 76.8%. Dergachyova et al. (2016) proposed a machine learning method. Specifically, they firstly described the input image by extracting the color, shape, and texture features of the image, and then they used several AdaBoost cascades for intermediate classification. Finally, a definite phase label is given by using the hidden semi-Markov Model. Based on visual features, the Acc of the model is close to 68%, and the Acc of fusion surgical instruments is close to 90%.The recent study in Dergachyova et al. (2016) is a method based on deep learning. The time smoothing convolution neural network and the classical HMM were used for phase recognition. The proposed network challenge is based on the residual network-200 pre-trained ImageNet, where the last layer is replaced by a new fully connected output layer, corresponding to 8 possible surgical phases. It was then fine-tuned on the M2CAI dataset using online data augmentation. The logarithmic probability output vector of the network was processed by temporal smoothing, and then passed to the HMM to correct possible classification errors for previously recognized frames. Twinanda et al. (2016) also proposed a method of deep learning based on pre-trained AlexNet, called PhaseNet, and they replaced the output layer and fine-tuned it using the M2CAI training dataset. At the second last layer of the PhaseNet, one-vs.-all linear SVM is obtained by using the image features extracted by CNN as input. Based on the Support Vector Machine classifier, the hierarchical HMM was introduced to reinforce the temporal constraint. The method was still based on two large datasets of laparoscopic cholecystectomy (Cholec 80 and EndoVis), which achieves better performance. The average Acc of offline analysis was highest, at 92.2% (Cholec80) and 86% (EndoVis), respectively. Shi et al. (2021) proposed a label-efficient Surgical workflow recognition method with a two-stage semi-supervised learning, named as SurgSSL which progressively leverages the inherent knowledge held in the unlabeled data to a larger extent. The SurgSSL method surpasses the state-of-the-art semi-supervised methods by a large margin.

## Materials and methods

**FIGURE 1:**
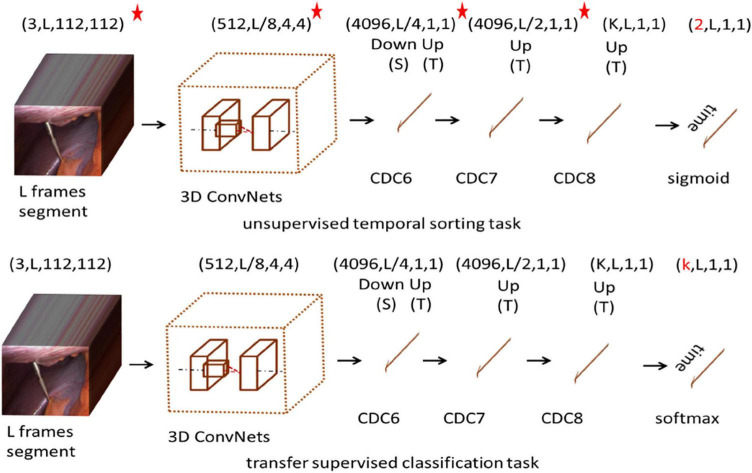
Architecture of the network. *The network is pre-trained and its parameters are fixed.

In this paper, we proposed a model for recognizing surgical workflow, as shown in Figure 1. Specifically, the top is an unsupervised time sorting task based on the CDC network, and the bottom is based on the top of the transfer supervised surgical phase classification task. The weights of the layers marked with a star can be passed. The first row shows the shape of the output data of each layer. First, the surgical video clip is fed into 3D ConvNets, and the temporal length is reduced from L to L/8. CDC6 has kernel size (4, 4, 4), Stride (2, 1, 1), padding (1, 0, 0), so the height and width are reduced to 1, while the temporal length increases from L/8 to L/4. CDC7 and CDC8 kernel size (4 1 1), Step (2, 1, 1), padding (1, 0, 0), so CDC7 and CDC8 further perform up-sampling in time by a factor of 2, so the temporal length is back to L in the unsupervised temporal sorting task, sigmoid layer is added on top of CDC8 to determine whether is correct for the order of the given L frames. In the transfer supervised classification task, a frame-wise softmax layer is added on top of CDC8 to obtain confidence scores for every frame. Each channel stands for one class, obtaining confidence scores for every frame.

### Unsupervised spatio-temporal context learning

In this section, we describe how to train the CDC network using unmarked video. We do this by addressing a task that requires the CDC to sort L given frames in the correct temporal order. For this, a large dataset from multiple surgical intervention is used. We assume that solving such a task requires CDC to learn to extract visual cues that describe the temporal flow of the surgical workflow.

The CDC (Shou et al., 2017) network is based on 3D convolution C3D network, which simultaneously carries out spatial convolution (for semantic abstraction) and temporal convolution (for frame-level resolution). It has a unique property in the spatio-temporal interactions between joint modeling and summarizing. The CDC network uses from conv1a to conv5b as the first part of the C3D network. For the remaining layers in the C3D, CDC keeps pool5 to perform max pooling in height and width by a factor of 2 but keeps the temporal length. The CDC sets the height and width of the network input as 112 × 112. Given an input video segment with a temporal length L, the output data shape of the pool5 is (512, L/8, 4, 4). To maintain the original temporal resolution (frame level), the CDC makes up-sampling in time (back to L from L/8) and down-sampling in space (from 4 × 4 to 1 × 1). More information is described in Shou et al. (2017).

**FIGURE 2:**
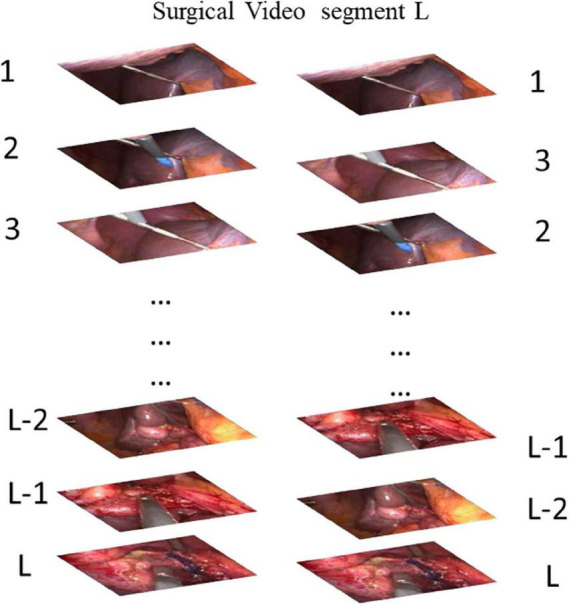
Our task for pretraining a CDC whether is the order of the given L frames correct? (Answer: The Left is correct).

Our CDC training tasks are shown in Figure 2: Given the same surgical video input for a video clip of temporal length L, what is the most relative order of L frames? That is, is the order of the given L frames correct? We uniformly sample L random frames from the video of the surgical intervention at the moment of transfer and enter them into our CDC. The transfer moment is shown in Figure 1. The CDC must calculate the relative order of L frames in the original video. That is, determines whether the given L frame is in the correct order? That is, in the last layer of the network, we have two categories of L frames, the correct order is positive, otherwise it is negative. We assume that solving this task requires the CDC to extract visual cues related to the surgical process in order to understand the temporal flow of surgical intervention. At the same time, the learning of temporal information is carried out in this process.

The total loss is defined as:


(1) $$\begin{array}{c} \text{L}=-\sum_{i}l\,a\,b\,e\,l_{i}*\log\left(p\,r\,o\,b_{i}\right)+\left(1-p\,r\,o\,b_{i}\right)*\log\left(1-p\,r\,o\,b_{i}\right) \end{array}$$

where *label*_*i*_ is the ground truth for i-th segment, *prob*_*i*_ is predictions for i-th segment.

When an unsupervised dataset is generated, data generation is primarily performed randomly at the time of conversion. Each phase is randomly sampled according to the ratio column, and the main sampling point is the transfer point. The specific sampling is related to the experimental dataset.

### Knowledge transfer for recognition of surgical phase

The phase sequence indicating a surgical process encodes some form of abstract knowledge about a given procedure. The knowledge can be extracted and utilized to improve various operations on surgical process data, including analysis, recognition and prediction. It is particularly assumed that the knowledge gained from one procedure can improve the prediction of the surgical phase of another procedure. The knowledge involved may include dependencies between phases in a sequence, relationships between elements in an activity, and connections between individual elements of different activities. In view of the difficulty of formalizing the concealment of knowledge, the CDC network can extract features from time and space at the same time, so the CDC network is chosen as a method to extract and transfer knowledge.

Deep neural networks have an interesting property that enables networks to store extracted information in a distributed hierarchical manner. It means that the basic information that is more common for many areas stored separately from the features that describe the characteristics of a particular domain. It also means that this information can be shared with other learning goal (e.g., other training task or area). In the deep model, the knowledge learned from the data is encoded into the weight matrix of the internal parameters of the representation layer. In order to establish the value of internal parameters, the domain containing a large number of training samples is first trained. Then, depending on the quantity and quality of data in the actual target domain, there are three transfer options. First, if the new data is close enough to the data used for training, and the task has not changed, we can use the same training model directly for the new data. The second option is to use the weights (in whole or in part) of the training model as the initialization of the new model. This applies where a reasonable amount of new data is available for training use. The third option, called fine-tuning, is typically used when the new domain contains only a small number of examples. It includes importing the trained weight matrix into the new model, but “freezes” some layers that usually contain more basic features during training. The weight setting of pre-training on other data is usually more optimized than random initialization. The network can benefit from what has been learned, thus, we should focus its “attention” on the specific characteristics of the new data. This section is based on the CDC time sorting network for knowledge transfer learning. Modify the final output layer of the CDC network to be L and classify each surgical step. In the transfer supervision classification task, the Softmax output is the vector of the *K*-value. Note that for the i-th class:


(2) $$\begin{array}{c} p_{n}^{i}\,\left[t\right]=\frac{e^{o_{n}^{\left(i\right)}\,\left[t\right]}}{\sum_{j=1}^{k}e^{o_{n}^{\left(i\right)}\,\left[t\right]}} \end{array}$$

The total loss L is defined as:


(3) $$\begin{array}{c} L=\frac{1}{N}\,\sum_{n=1}^{N}\sum_{t=1}^{L}\left(-\log\left(P_{n}^{\left(z_{n}\right)}\,\left[t\right]\right)\right) \end{array}$$

Where *z_n_* is the ground truth class label for the n-th segment.

## Experiment and result

### Dataset and data sampling

**FIGURE 3:**
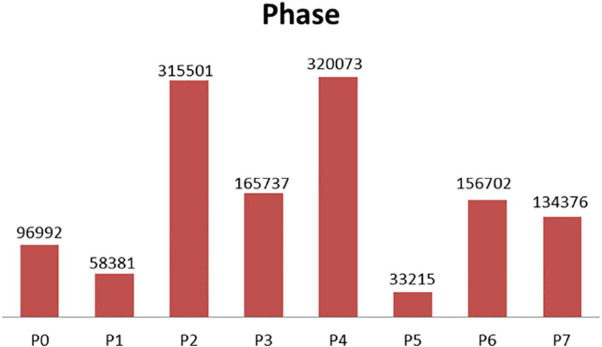
Phase distribution (training data at 1 fps).

The experiment in this paper is based on the M2CAI16-workflow dataset, which is available from http://camma.u-strasbg.fr/m2cai2016/. It contains videos of 41 cholecystectomy processes from the University Hospital of Strasbourg/IRCAD (Strasbourg, France) and Klinikum Rechts der Isar Hospital (Munich, Germany). The datasets are divided into two parts: the training subset (containing 27 videos) and the testing subset (14 videos). The videos are recorded at 25 fps. All the frames are fully annotated with 8 defined phases: (1) trocarplacement, (2) preparation, (3) calot triangle dissection, (4) clipping and cutting, (5) gallbladder dissection, (6) galbladder packaging, (7) cleaning and coagulation, and (8) gallbladder retraction. The list of phases in the dataset is shown in Table 1. The distribution of the phases in dataset is shown in Figure 3.

**TABLE 1:** List of phases in the dataset.

ID	Phase
P0	Trocar placement
P1	Preparation
P2	Calot triangle dissection
P3	Clipping and cutting
P4	Gallbladder dissection
P5	Galbladder packaging
P6	Cleaning and coagulation
P7	Gallbladder retraction

**FIGURE 4:**
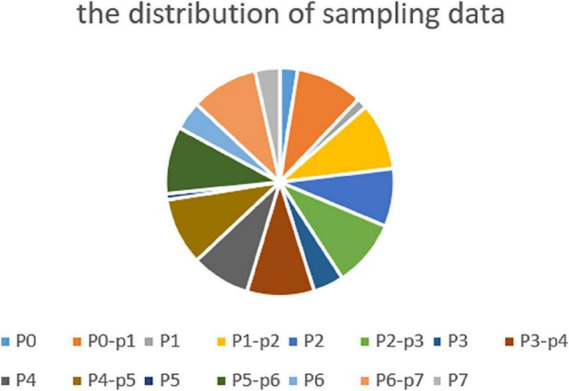
Sampling data at all phases and transitional moment.

In the case of a frame rate of 1, a total of 1.3 million frames are available. Depending on the distribution of the surgical phase, we randomly collected 250,000 surgical video clips from different surgical phases, 500,000 surgical video clips for the transition period, and 750,000 surgical video clips for unsupervised temporal learning. The sampling data for each stage and transition time is shown in Figure 4.

### Comparison algorithms

We compared our method with several state-of-the-art method. Dergachyova et al. (2016) and Twinanda et al. (2016) are two of the methods submitted to the M2CAI 2016 challenge. CNN-biLSTM-CRF (Yu et al., 2019) is a semi-supervised method with 12 labeled vides and 15 unlabeled videos. The cnn-lstm-net and spatial-net are temporal and spatial models depicted in Chen et al. (2018). In the CAE method (Qi et al., 2020), a convolutional auto-encoder network is trained first, and then surgical process segmentation is performed.

### Metrics and result

**FIGURE 5:**
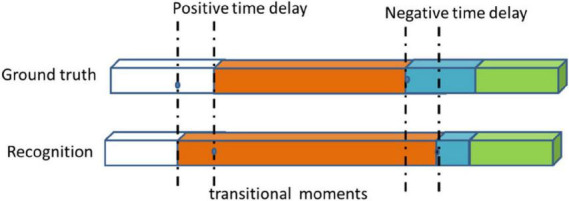
Examples of negative and positive transitional delays and transitional moment.

As described in other literatures (Chen et al., 2018; Qi et al., 2020; Shi et al., 2021), the metrics includes standard accuracy (Acc), recall rate (Rec), precision (Pres), average conversion delay (ATD), and real transition ratio (TRR). Some applications do not require a frame-by-phase identification. They may tolerate a certain time delay, but have no fundamental impact on the assistance provided. We introduced the concept of a transition window that a time interval centered on a real transitional moment, at both ends, authorizing an acceptable delay *d*. If the time moment being checked is in the transition window and occurs because of a delay, it is considered true. In this experiment, we set up different delay time *d* to calculate the Acc, Rec, and Pres of the model. We called it a time delay standard score. ATD measures the latency generated during all conversions of all available interventions in order to make an average estimate of the delay (see Figure 5). The negative and positive delays are measured separately and used to define the range of values for the average transition delay. A negative delay indicates that the transition between phases is detected in a delayed manner with regards to the ground truth. Conversely, positive delay means that the system decides to switch phases prematurely before the actual transition, details in Dergachyova (2018). The TRR Metric calculates the actual TRR detected between numbers. It is an indicator of system stability and reflects the robustness of the system, as systems with high TRR may have a lower tolerance for intrinsic changes in input data. This ratio also provides a simple and intuitive idea of how many incorrect transfer moments are detected with the number and actual number of transitional moments that they actually detect (see Equation 4).


(4) $$T\,R\,R=\frac{s^{{}^{\prime }}}{s}$$

where the *s* is the real transfer moment, the *s’* is transfer moment detected by the model.

Based on the data collected randomly, we first carry out unsupervised temporal task learning, pre-training, and then use the transfer learning method to carry out phase supervision classification. The corresponding results are shown in Tables 2, 3.

**TABLE 2:** ATD, TRR metrics for phase recognition.

Methods	ATD	TRR
Ours	[−15 s; 30 s]	6.0
Twinanda	[−23 s; 54 s]	3.8
Dergachyova	[−45 s; 70 s]	2.7

**TABLE 3:** Time delay standard scores metrics for phase recognition.

Methods	Scores			*d* = 30 s			*d* = 60 s		
			
	Acc	Rec	Pres	Acc	Rec	Pres	Acc	Rec	Pres
Ours	**89.2**	**76.5**	**78.3**	**90.6**	**77.8**	**80.9**	**91.4**	**78.9**	**82.5**
Twinanda	75.2	64.6	69.0	80.5	70.6	77.8	82.9	74.9	79.5
Dergachyova	68.6	60.9	64.1	72.1	65.3	66.2	76.6	71.4	78.1

Bold values indicate the optimal result in the algorithm comparison.

As can be seen from the results in Table 2, our approach has the shortest transition delay [−15s; 30s]. As can be seen from the results in Table 3, the standard Acc, Rec, and Pres of our model reach 89.2, 76.5, and 78.3%, respectively. Based on these results, this is why our model improves Acc less than other usage time delay standard scores. Our approach is more suitable for applications that require rapid system response. However, it makes too many incorrect conversions between phases (6 times more than it should be). On the other hand, the Dergachyova method provides greater delays recognition, but less incorrect phase change peaks (TRR = 2.7). Compared with our method, its recognition is more consistent. The Twinanda method also has a lower TRR. This shows that our model is more suitable for online use, while the Twinanda method and the Dergachyova method are suitable for offline use. The results in Table 3 show how to use the delay transition window to improve performance scores. This helps to make a clearer estimate of how close these methods are actually to clinical applications in specific applications. From the above analysis, it is also important that we do not use a single indicator to distinguish and objectively compare these surgical phases of the identification model. In Table 4, the experimental results of Rec and Pres with no time delay are compared. The results show that our method outperform the comparison methods.

**TABLE 4:** Comparison results with no time delay.

Methods	Rec	Pres
Dergachyova	60.9	64.1
Twinanda	64.6	69.0
CNN-biLSTM-CRF	69.9	74.5
Cnn-lstm-net	72.2	60.8
Spatial-net	72.9	73.4
CAE	68.3	72.7
**Ours**	**76.5**	**78.3**

Bold values indicate the optimal result in the algorithm comparison.

## Conclusion

The automatic recognition of the current surgical phase can provide the correct computer assistance at the right time, which is the basis of realizing the context-aware OR system. However, the lack of clinical data in this area is a well-known problem. This creates obstacles to the recognition and analysis of surgical workflow tasks that require significant amounts of data. In this paper, an unsupervised CDC network method is proposed, which simultaneously carries out spatial convolution (for semantic abstraction) and temporal convolution (for visual resolution) of surgical workflow frame sequences. Then through the transfer learning, the CDC network is fine-tuned to classify the operative stage. Based on M2CAI 2016 challenge dataset, experiments and comparisons have been made, and good results have been obtained. The transparency is a very important attribute of the medical system. In this paper, we use a deep learning method has been criticized for the nature of its learning process that is poorly understood. This can cause distrust among doctors. In the future work, we want to visualize the learning processes of deep networks in order to understand exactly what they have learned.

## Data availability statement

Publicly available datasets were analyzed in this study. This data can be found here: https://mldta.com/dataset/m2cai-2016-challenge/.

## Author contributions

Y-WC and JZ: study concept and design. K-HZ, Y-WC, and PW: analysis and interpretation of data. Y-WC, Z-YH, and PW: technical support. Y-WC: obtain funding. Y-WC, K-HZ, and Z-YH: writing original manuscript. K-HZ and Y-WC: revision of manuscript. All authors contributed to the article and approved the submitted version.
